# Supplementary material for: Deletion of wfs1 Impairs Oligodendrocyte Precursor Cells Dorsal Distribution and Myelination Through the wfs1-hmgcs1 Axis in Zebrafish
Source: Biology (Basel). 2026 Aug 16;15(16):1402. doi: 10.3390/biology15161402 (PMC13510151; doi:10.3390/biology15161402)
Supplement: Supplementary file 1 [file biology-15-01402-s001.zip › File S1-Transcriptome sequencing data and differentially expressed genes (DEGs) in wfs1-- zebrafish at 5 dpf/2.KEGG/wfs1KOvsWT/ALL/src/dre04130.html]

dre04130


- dre:565822

- Up regulated genes

565822\_1(4.81847946831565)

- dre:393294

- Down regulated genes

393294(-1.09658675379848)

- dre:436605

- Up regulated genes

436605(8.54393697549803)

- dre:436605

- Up regulated genes

436605(8.54393697549803)

- dre:393836

- Up regulated genes

393836(1.13083822437546)

- dre:567385

- Up regulated genes

567385\_1(3.31538730941156)

- dre:325484

- Up regulated genes

325484(1.14992821569271)

- dre:571872

- Up regulated genes

571872(1.72813109564786)

- dre:571872

- Up regulated genes

571872(1.72813109564786)

- dre:449923

- Up regulated genes

449923(3.08455507116647) 449923\_1(1.39798369726066)

- dre:393236

- Up regulated genes

393236(3.17856048240125)

- dre:567385

- Up regulated genes

567385\_1(3.31538730941156)

Close
